# Anti-SARS-CoV-2 Antibody Testing: Role and Indications

**DOI:** 10.3390/jcm12247575

**Published:** 2023-12-08

**Authors:** Sylvia Mink, Peter Fraunberger

**Affiliations:** 1 Central Medical Laboratories, 6800 Feldkirch, Austria; 2 Private University in the Principality of Liechtenstein, 9495 Triesen, Liechtenstein

**Keywords:** COVID-19, booster vaccination, anti-SARS-CoV-2 antibodies, SARS-CoV-2, antibody testing, correlate of protection

## Abstract

Since the onset of the COVID-19 pandemic in March 2020, over 769 million confirmed COVID-19 cases, including close to 7 million COVID-19-related deaths, have been reported. Although mortality rates have dropped notably compared to the first months of the pandemic, spikes in reported cases and mortality rates continue to be registered. Both recent spikes in case numbers and the continued emergence of new variants suggest that vulnerable patient groups, including older adults, immunocompromised patients, and patients with severe comorbidities, are going to continue to be affected by COVID-19. In order to curb the pandemic, relieve the pressure on primary care facilities, and reduce mortality rates, global vaccination programs have been established by the WHO, with over 13.5 billion vaccine doses having been administered globally. In most immunocompetent individuals, vaccination against COVID-19 results in the production of anti-SARS-CoV-2 spike antibodies. However, certain patient subsets have inadequate or reduced immune responses, and immune responses are known to decrease with age. General recommendations on the timing of booster vaccinations may therefore be insufficient to protect vulnerable patients. This review aims to evaluate the clinical role of anti-SARS-CoV-2 antibodies, focusing on measurement indications, prognostic value, and potential as a correlate of protection to guide future booster vaccination strategies.

## 1. Introduction

Since the official beginning of the COVID-19 pandemic in March 2020, over 769 million confirmed COVID-19 cases, including close to 7 million COVID-19-related deaths, have been reported to the World Health Organization (WHO) [1]. Infection fatality rates were highest in vulnerable patient subsets such as older adults, with age having been recognized as an independent risk factor for mortality in COVID-19 [2,3]. Between 2020 and 2023, approximately 10% of deaths in the United States of adults aged 50 years or older were attributed to COVID-19 [4]. Even in non-elderly patients, infection fatality rates increased up to 4-fold for every decade [5].

Although mortality rates have dropped notably compared to the first months of the pandemic, spikes in reported cases and associated mortality rates continue to be reported [1]. In addition, EG.5, a sublineage of XBB.1.9.2 and a new variant of interest, has triggered a global increase in infection numbers [6,7]. Both recent spikes in case numbers and the continued emergence of new variants [6] suggest that vulnerable patient groups, including older adults [2], immunocompromised patients [8,9] and patients with severe comorbidities [10,11,12,13], are going to continue to be affected by COVID-19.

In order to curb the pandemic, relieve the pressure on primary care facilities, and reduce mortality rates, global vaccination programs have been established by the WHO [14]. To date, close to 13.5 billion vaccine doses have been administered globally, with approximately 66% of persons vaccinated with a complete primary series and over 31% having received at least one booster dose [15]. In the majority of immunocompetent individuals, vaccination against COVID-19 results in the production of anti-SARS-CoV-2 spike antibodies [16,17].

This review aims to evaluate the role of anti-SARS-CoV-2 antibodies in a clinical setting, with a special focus on indications for measuring anti-SARS-CoV-2 spike antibodies, their prognostic value, and their potential usefulness as a correlate of protection for guiding future booster vaccinations.

## 2. Viral Structure

Coronaviruses are enveloped viruses that possess a single-stranded RNA genome and are characterized by high genetic recombination and mutation rates [18]. SARS-CoV-2 belongs to the genus of betacoronavirus and shares approximately 80% similarity with the genomic sequence of SARS-CoV. The genome of SARS-CoV-2 is close to 30 kb in length and encodes the structural proteins nucleocapsid (N), spike (S), membrane (M), and envelope (E), which are important for virion assembly and suppressing of the host immune response [19].

The S protein is a heavily glycosylated homotrimer and belongs to the type I membrane protein family. The protein is situated on the outer surface of the virion and comprises two functional subunits, S1 and S2. The S1 subunit contains a receptor-binding domain (RBD) that is able to recognize and bind the human angiotensin-converting enzyme 2 (ACE2) receptor on host cell membranes, which triggers the fusion of viral and host cell membranes [20].

After entry into the host cell, the E protein regulates viral lysis and the subsequent release of the viral genome into the cell. The N protein constitutes an important structural component inside the virion that protects and packages the viral genome [19].

## 3. Antibody Response to Infection

Following SARS-CoV-2 infection, the majority of immunocompetent individuals develop an adaptive immune response via B and T cell-mediated immunity [21,22]. The humoral response in humans includes the production of antibodies that target the domains of N and S proteins. IgM, IgG and IgA antibodies can be detected in peripheral blood samples within one to four weeks after infection [23,24]. The estimated median time to seroconversion was 10 to 12 days from symptom onset and was comparable across all antibody isotypes [23]. Neutralizing antibodies are able to inhibit viral replication in vitro. Most neutralizing antibodies against SARS-CoV-2 target the RBD of the S1 subunit [19], and RBD-specific IgG titers correlate with neutralizing potency [25]. RBD-specific IgG responses were detected as early as 6 days after PCR confirmation and 8 days after symptom onset [25]. Other targets for antibodies include the N-terminal domain (NTD) of the S1 subunit and the S2 region. However, most of these antibodies were found to exhibit limited or no neutralizing activity [26,27,28].

Antibody responses were stronger in critically ill patients [24], and seroconversion was estimated to occur 4 days earlier in hospitalized patients compared to non-hospitalized patients, regardless of isotype [23]. Seroreversion, the return to becoming seronegative, occurred earlier for IgA and IgM than IgG, with a median time of 49 days for IgM and 71 days for IgA [23]. In contrast, IgG antibodies were shown to persist for several months in most patients [29], although the precise duration remains unclear. One study reports sustained anti-S1 IgG seropositivity in 68.6% of patients 12 months after symptom onset [30]. Loss of seropositivity seems to occur earlier in patients with milder courses of COVID-19 [31]. Contrarily, patients with severe courses exhibit higher antibody titers and longer persistence of detectable antibodies [32].

Antibody production is highly variable between individuals [33,34,35,36,37,38,39,40], and some patients remain seronegative. For instance, a study of more than 2500 health care workers in the United States found that 6.3% were seronegative at least 2 weeks after the onset of symptoms [41]. Risk factors for lack of antibodies include asymptomatic or mild disease and immunosuppressive therapy [41,42]. In addition, the sensitivity for detecting seroprevalence varies by assay, which may cause an increasing number of seronegative results with less sensitive assays as time passes and antibody levels begin to decline following the infection [43].

## 4. Types of Antibody Tests

Two broad categories of antibody tests can be differentiated—neutralizing antibody detection tests and binding antibody detection tests. Neutralizing antibody detection tests, also known as viral neutralization tests, determine whether antibodies from serum or plasma samples are able to inhibit viral growth of SARS-CoV-2 in cell cultures. Viral neutralization tests, such as the plaque-reduction neutralization test and microneutralization test, either employ SARS-CoV-2 or recombinant SARS-CoV-2 that expresses reporter proteins (the S protein, for instance). They are time-consuming and may take several days to complete, making them unsuitable for high-throughput testing. Routine laboratories are normally not equipped to perform viral neutralization tests because they require a viable virus and therefore a higher biosafety level (BSL) classification.

Pseudovirus neutralization tests do not rely on SARS-CoV-2 but use less harmful recombinant pseudoviruses that express the S protein of SARS-CoV-2. Commonly employed pseudoviruses are the vesicular stomatitis virus or lentivirus [44]. Depending on the exact strain used by these tests, they may also be performed in laboratories with lower biosafety level classifications, including BSL2.

In contrast, binding antibody detection tests do not rely on a viable virus but use purified proteins of SARS-CoV-2 only. The basic principle of antibody detection consists in the incubation of viral proteins with patient serum or plasma to capture specific antibodies that are present in the sample material [45]. These tests can be automated, have short turn-around times, and are widely available in routine laboratories. Different platforms are currently in use, including enzyme-linked immunosorbent assays (ELISA), chemiluminescent microparticle immunoassays (CMIA), lateral flow immunoassays (LFA), and chemiluminescent immunoassays (CLIAs) [46]. Antigenic targets used for the detection of antibodies are primarily derived from SARS-CoV-2 N and S proteins. Antibodies against N protein domains are only detectable after infection with SARS-CoV-2, whereas antibodies against S protein domains may be present after an infection and/or after vaccination against SARS-CoV-2 [16,17]. Assays for the detection of antibodies against S protein employ either full-length or partial antigens. Full-length antigens consist of S1 and S2 subunits, whereas partial antigens utilize either S1 or receptor-binding domains (RBD). Of these, antibody titers against RBD are reported to correlate well with neutralizing potency [25]. The high correlation between RBD antibody titers and neutralizing antibodies, coupled with short turn-around times and wide availability, has made RBD antibody testing an attractive and feasible substitute for neutralization tests in clinical practice.

Different surrogate neutralization tests, or protein-based pseudo-neutralizing antibody assays, have been developed in which cells are replaced by receptors and the virus is replaced by surface proteins [44]. For instance, Johnson et al. [47] described a multiplexed solid-phase chemiluminescence assay that allows the simultaneous detection of IgG binding to four distinct SARS-CoV-2 antigens and quantification of antibody-induced inhibition of ACE-2 receptor binding. Surrogate neutralization tests are ELISA-based, do not use a viable virus, and therefore can be conducted by routine laboratories [44].

More than 50 test systems have been approved by the U.S. Food and Drug Administration (FDA). Their test characteristics, including antibody isotype detected, sensitivity, specifity, and positive and negative predictive values, are reported on the FDA website [46]. This variety of test systems makes comparisons, and interpretation of study results, based on different test systems more difficult and also limits the applicability of results in clinical practice [48]. Widely available, quantitative binding antibody assays that are standardized to an international standard are necessary to allow for the easier interpretation of studies on kinetics and duration of antibody response, correlation to neutralizing antibodies, and association of antibody levels with protection from COVID-19 and outcome, and to facilitate clinical application of these results [49].

An important step in this direction was taken by establishing the WHO international standard for anti-SARS-CoV-2 immunoglobulin, which allows reporting assay results in reference to an international standard of pooled human plasma from convalescent patients. This international standard is set at 250 international units (IU) of neutralizing activity per ampoule, which corresponds to 1000 binding antibody units (bau) per mL for binding assays. Using this standard, inter-laboratory variation was reduced more than 50 times for neutralization and more than 2000 times for ELISA assays [50].

## 5. Antibodies and Immunity from Infection

Several studies have reported that the presence of anti-SARS-CoV-2 antibodies notably reduces the likelihood of subsequent infection with SARS-CoV-2, although antibodies do not appear to confer full protection [51,52,53,54]. A study of 12,541 British health care workers showed that the presence of anti-spike or nucleocapsid antibodies was associated with a substantially reduced risk of SARS-CoV-2 reinfection in the ensuing 6 months. The adjusted incidence rate ratio for subsequent infection was 0.11 for participants with a positive antibody test compared to seronegative participants [51]. Similarly, an observational study from the U.S. that analyzed over 3 million individuals concluded that seropositivity was associated with 90% lower infection rates 90 days or more after first testing [52]. Concordantly, a large British study including over 30,000 participants indicated that a previous infection with SARS-CoV-2 lowered the risk of reinfection by 84% over a 7-month period after primary infection [55].

After 2-dose vaccination with mRNA-1273, antibody levels determined with anti-spike-IgG, anti-RBD-IgG, and two types of pseudovirus neutralization tests, were all inversely associated with COVID-19 risk and directly associated with vaccine efficiency [56]. Antibody measurements were conducted at day 29 and day 57, and the risk of COVID-19 occurrence was assessed over approximately 4 months following the second vaccine dose. COVID-19 risk increased incrementally with decreasing antibody levels. Estimated COVID-19 risk was about 10 times higher for participants with negative antibody levels compared to participants with antibody levels in the top 10%.

Accordingly, several therapeutic monoclonal antibodies have been shown to effectively neutralize infection with SARS-CoV-2 by blocking the RBD of the S protein, which prevents interaction of SARS-CoV-2 with the human ACE-2 receptor [26]. For instance, treatment with bamlanivimab, a neutralizing monoclonal IgG1κ antibody that was derived from screening antigen-specific B cells from a convalescent COVID-19 patient, significantly reduced the incidence of COVID-19 in the prevention group compared to placebo during a phase 3 trial conducted in the U.S. [57,58]. Other targets for monoclonal antibodies have also been explored, including the N-terminal domain, which is also located on the S1 subunit, the S2 fragment, or the S-trimer complex. However, most of these antibodies were found to lack neutralizing activity [26].

Antibodies may develop in response to vaccination or after infection with SARS-CoV-2 and are therefore tailored to the vaccine or the respective viral variant. The emergence of structural variations in new viral variants may affect the specifity and neutralization activity of existing antibodies. With regard to the Omicron variant, neutralizing antibody responses from reference sample pools were found to have a substantially lower neutralizing potency against the Omicron variant that against wild-type SARS-CoV-2 [33,59]. In spite of the extensive evasion of neutralizing antibodies, sufficiently high antibody titers were still deemed protective.

The effectiveness of higher antibody levels in clinical settings is also shown by the results from studies on booster vaccinations against COVID-19. In a large Israeli study encompassing over one million individuals aged 60 years or above, rates of confirmed infection were reported to be 11 times lower at least 12 days after booster vaccination compared to the non-boostered control group [60]. Other studies report similar results for younger patients, indicating restored vaccine effectiveness and lower infection rates after administration of booster doses [61,62,63].

Thus, the duration of protection from infection is likely associated with the antibody levels that were initially achieved after infection or vaccination, the decrease in antibody levels over time, and the administration of booster vaccinations [52]. However, there is currently no clear indication how high antibody levels need to be in order to provide protection from infection. This level may further be dependent on the overall physical condition of the individual and the function of the cellular immune system.

## 6. Association with Severity and Outcome

Apart from infection rates, antibody levels have also been shown to be associated with the severity and mortality of COVID-19. Khoury et al. [64] reported that antibody levels were highly predictive of protection from symptomatic COVID-19. Of note, the neutralization levels required for protection against detectable infection were higher than the neutralization levels needed to protect against severe infection. These findings are corroborated by a British study examining the correlation between antibody levels and symptomatic infections 28 days after a second vaccination dose, which found that higher antibody levels were associated with a reduced risk of symptomatic infections [65]. A vaccine efficacy of 80% against symptomatic infection was achieved above 506 binding antibody units for anti-spike and anti-RBD antibodies.

Another study from Qatar comprising over 2.2 million persons that had received at least two doses of either BNT162b2 (Pfizer-BioNTech) or mRNA-1273 (Moderna) noted that an additional dose provided some protection against symptomatic COVID-19 infections, although the effect was smaller in patients infected with Omicron compared to the Delta variant [66]. In contrast, for both the Omicron and Delta variants, a booster dose conferred a high degree of protection against COVID-19-related hospitalization and death. Similarly, another large cohort study involving 1.1 million older adults found that a booster dose lowered the rate of severe illness by a factor of 19.5 (95%CI 12.9 to 29.5) [60]. In this study, severe disease was defined as a resting respiratory rate of more than 30 breaths per minute, an oxygen saturation of less than 94% without oxygen administration, or a ratio of partial pressure of arterial oxygen to fraction of inspired oxygen of less than 300. Comparable results were found after application of a fourth vaccine dose, with participants exhibiting 3.5 times lower adjusted rates of severe COVID-19 after having received a fourth dose, compared to participants who had only received three doses [67]. While protection against infection waned quickly, protection against severe disease was present during the 6 weeks of follow-up after the fourth dose.

In a study of approximately 800,000 patients aged 50 years or older, mortality rates were 90% lower in those who had received a booster vaccination compared to those who had not (0.16 deaths per 100,000 persons per day versus 2.98 deaths per 100,000 persons per day; adjusted hazard ratio 0.10, 95%CI 0.07–0.14, *p* < 0.001) [68].

We previously reported that anti-SARS-CoV-2 spike antibodies measured on hospital admission of patients with COVID-19 were strongly inversely associated with in-hospital mortality in both vaccinated and non-vaccinated patients [69]. For patients infected with the currently prevailing Omicron variant, risk of in-hospital mortality was 4 times higher if anti-SARS-CoV-2 antibody levels were below 1200 U/mL (aOR 4.08, 95%CI 1.81–9.20, *p* < 0.001) [69].

While there was a clear association between booster doses and reduced rates of severe COVID-19 and mortality, many of the studies on vaccine efficacy did not report antibody levels. The question whether those individuals that did experience severe courses despite booster vaccinations had lower levels of neutralizing antibodies is of particular interest. Data with regard to this issue may be useful for determining antibody cutoff levels that are associated with a high degree of protection, to help guide future recommendations on the timing of booster vaccinations. Relevant publications on the role of antibodies as a correlation of protection are summarized in Table 1.

## 7. Reduced Antibody Response in Older Adults

Certain subsets of the general population have been found to be particularly susceptible to severe courses of COVID-19 and COVID-19-related mortality. Independent risk factors that have been identified are old age, receiving immunosuppressive therapy, and a number of pre-existing comorbidities, including type II diabetes, obesity, cardiovascular and renal diseases [2,11,12,13,70].

Older adults have been particularly hard-hit by the pandemic, with advancing age being directly associated with elevated risks of hospital admission, critical illness, and COVID-19-related mortality [2,3,71,72,73]. A large international study comparing data from 29 countries reported that, even for patients aged 70 years or younger, infection fatality rates increased by a factor of four with each passing decade [5]. Comparing seroprevalence data from New York, England, Switzerland, Sweden, Belgium, Mexico, and Brazil, Rickards et al. [74] estimated that infection fatality rates rose by 3–4 times for every 20 years’ increase in age, which constitutes a slightly lower but still notable increase in risk. Accordingly, the higher the percentage of persons aged 65 years or above in a population, the higher were the recorded average weekly fatality rates due to COVID-19 [75].

The reasons behind this substantially increased risk are thought to be a combination of general frailty, the increasing prevalence of severe comorbidities with age [76], and a loss of function of both the adaptive and the innate immune systems [77].

Old age is associated with a series of alterations to the adaptive immune system, including an accumulation of aberrant B cells, reduced T cell function, and a decrease in humoral immune responses [78,79,80]. Although antibody production varies considerably between individuals, the quality, strength, and durability of vaccine-induced immune responses generally decline with age [26,34,35,37,38,39,77,81]. Bone marrow degeneration in old age leads to a reduction in the production of naïve B lymphocytes [82]. While the peripheral plasma cell counts may remain unaltered, a significant proportion of these cells has previously encountered an antigen. This prior exposure restricts their capacity to bind new antigens. Consequently, these factors contribute to a reduced and delayed production of antibodies that target new epitopes [83]. In addition, antibody affinity decreases with age, primarily stemming from lower rates of isotype switching, diminished somatic hypermutation, and a decrease in spontaneous mutations in variable regions [84,85].

Nonetheless, studies show positive results for timely booster vaccinations in older adults. For instance, an Israeli study comprising over one million participants reported that booster vaccinations against COVID-19 were associated with a significantly lower risk of severe illness in older adults aged 60 years or above [67]. Thus, timely and regular administration of booster vaccinations is critical to reduce rates of severe illness and COVID-19 mortality in older adults [64,86].

Considering the reduced immunogenicity of vaccines, the reduced quality and lower persistence of humoral responses in older adults, as well as the wide variations in antibody responses between individuals, monitoring antibody levels after vaccination would be useful to ensure the efficacy of vaccines and protect this vulnerable patient group.

## 8. Indications for Antibody Testing

While antibody testing is not a replacement for direct virological testing with antigen detection or PCR-based tests during the acute phase of an infection, measuring antibody levels may be useful in a number of situations. An overview of indications for anti-SARS-CoV-2 antibody testing is given in Table 2.

Nucleocapsid antibodies may be used to confirm a preceding infection with SARS-CoV-2, if a direct virological test was not performed during the infection and if COVID-19-related complications or post-acute sequelae due to COVID-19 are suspected [87]. However, at present, measuring anti-nucleocapsid antibody levels does not allow an estimation of how long ago an infection may have occurred. As anti-SARS-CoV-2 spike antibodies are formed after vaccination against COVID-19, these types of antibodies are only useful for confirmation of a preceding infection if the individual has not previously been vaccinated against COVID-19.

In the unlikely case that direct virological testing failed to provide a positive result, seroconversion from negative to positive antibody levels may indicate a SARS-CoV-2 infection between the dates when the samples were obtained.

Another application for anti-SARS-CoV-2 antibody testing is in conducting seroprevalence studies for epidemiological or public health management purposes [87]. In this context, antibody testing may in theory be used to retrospectively determine the size of an outbreak, the extent to which an infection has spread in a certain population, to estimate the prevalence of mild and asymptomatic infection, to calculate infection fatality rates and vaccine effectiveness, and to estimate the proportion of the population who may be protected against infection or severe courses. Quantitative serological assays with high sensitivity are required to assess the development of antibody levels over time and to examine the anticipated corresponding decrease in protection from infection, severe disease, and mortality [87,88]. Considering the high global vaccination coverage [14], and the corresponding increase of seropositive individuals with anti-SARS-CoV-2 spike antibodies, anti-nucleocapsid antibodies need to be employed in order to determine the spread of infection and the prevalence of mild or asymptomatic disease. However, the usefulness of antibody testing for epidemiological indications such as registering the size of new outbreaks has substantially declined and will continue to further diminish over time, as persons who have had previous contact with SARS-CoV-2, and are thus already seropositive for anti-nucleocapsid antibodies, cannot be differentiated from new cases using anti nucleocapsid or anti-SARS-CoV-2 antibodies. Further, individuals who have received vaccines based on an inactivated virus instead of spike-protein-based vaccines cannot be differentiated from individuals who have previously undergone SARS-CoV-2 infection.

Monitoring antibody responses at population level may be useful for determining the efficacy of vaccines against novel variants of concern or variants of interest. Applications include assessing the proportion of individuals who form neutralizing antibodies against a new variant after vaccination, determining the strength of the antibody response, and its duration [16]. However, repeat measurements before and after vaccination may be necessary to assess the change in antibody levels in individuals who are already seropositive at the time of vaccination. 

Patient groups who are unable to form an adequate antibody response, such as immunosuppressed or oncological patients, are particularly susceptible to severe courses of COVID-19 [8,9,89]. In these patient groups, measuring antibody levels may be useful to identify seronegative patients who would benefit from therapy with monoclonal antibodies against SARS-CoV-2, such as casirivimab or bebtelovimab [87,89]. 

In addition, antibody testing may be used to monitor antibody level decline after administration of monoclonal antibodies in patients with prolonged immunosuppression who might require additional doses.

Anti-SARS-CoV-2 antibody levels are associated with protection against severe courses and COVID-19-related mortality [64,69]. However, antibody production in response to COVID-19 infection or vaccination is highly variable and depends on the individual patient, which may result in varying degrees of protection after booster vaccinations [33,34,35,36,37,38,39,40].

Current recommendations for booster vaccinations by the WHO [90] vary between 6 to 12 months for the highest-risk groups, comprising older adults and younger adults with significant comorbidities or severe obesity. While these recommendations include shorter intervals for higher-risk groups, the variability of immune responses between individuals is not taken into account. As of May 2023, the CDC currently recommends that people aged 65 or older may receive one additional booster dose at least 4 months after the first dose of a bivalent mRNA vaccine and does not recommend additional booster vaccinations for adults aged between 12 and 65 years after one dose of a bivalent mRNA vaccine [91].

However, certain patient subsets have inadequate or reduced immune responses, and immune responses are known to decrease with age [77]. General recommendations on the timing of booster vaccinations may therefore be insufficient to protect vulnerable patients. Measuring antibody levels in these patient groups would allow the identification of patients who have not formed an adequate antibody response and thus remain at high risk of severe disease and mortality. Offering additional or more frequent booster vaccinations may help protect these patients.

While there appears to be an incremental increase in protection with increasing antibody levels, defining thresholds for protection may also be useful in clinical practice to stratify patients according to risk of severe disease and mortality and to advise the timing of booster recommendations. We have previously shown that anti-SARS-CoV-2 spike antibody levels above a threshold of 1200 U/mL are associated with markedly reduced mortality in the general population [69]. Providing such a point of orientation would be useful in clinical practice to inform decisions on whether to recommend additional booster vaccinations to a patient. Nonetheless, data on potential cutoffs remains limited, and the threshold mentioned above needs to be confirmed with additional studies.

## 9. Conclusions

Anti-SARS-CoV-2 antibody testing is useful for a range of indications, most notably for evaluating seroprevalence to determine vaccine efficacy against emerging variants and to establish appropriate public health measures, for assessing the prognosis of patients infected with COVID-19, for identifying patients who would benefit from treatment with monoclonal antibodies against SARS-CoV-2, and potentially for guiding the timing of booster vaccinations in vulnerable patient groups.

A threshold of 1200 U/mL has been suggested [69] as a primary point of orientation to help inform decisions on additional booster vaccinations in an outpatient setting. However, more data is needed for defining protective antibody thresholds.

## 10. Future Outlook

Given the recurring spikes in COVID-19 cases and the high genomic variability of SARS-CoV-2, new variants of concern/interest are likely to emerge. No lifelong immunity has been observed in other endemic, seasonal coronaviruses [92], and current data indicate that neither infection nor vaccination confers durable immunity against SARS-CoV-2 or protection from severe disease and mortality. Although infection fatality rates have declined notably compared to the onset of the pandemic, vulnerable patient groups including older adults, patients with severe comorbidities and/or obesity, and immunocompromised patients remain at risk.

Thus, vaccination strategies aimed at effectively protecting these groups in the coming years are required. To ensure both efficiency and efficacy, these strategies would ideally be based on a correlate of protection. The evaluation of SARS-CoV-2-specific antibody responses at population level will be critical for determining public health measures aimed at reducing transmission, COVID-19-related morbidity and mortality, and alleviating the pressure on health care facilities in case a new variant of concern emerges.

In a clinical setting, anti-SARS-CoV-2 antibody testing may be required analogously to Hepatitis B titers as proof of effective vaccination for staff working with vulnerable patient groups, such as older adults or immunocompromised patients.

## Figures and Tables

**Table 1:** Overview of main human studies on the association of anti-SARS-CoV-2 antibodies with immunity from infection and COVID-19 severity.

Author	Title	Year	*n*	Study Type	Study Population	Main Result
**Immunity from infection**						
Lumley [51]	Antibody Status and Incidence of SARS-CoV-2 Infection in Health Care Workers.	2021	12,541	cohort study	health care workers	anti-spike/nucleocapsid antibodies were associated with reduced risk of reinfection
Harvey [52]	Association of SARS-CoV-2 Seropositive Antibody Test with Risk of Future Infection.	2021	3,257,478	cohort study	de-identified patients with antibody test results from commercial health data sources	seropositivity is associated with protection from infection
Jeffery-Smith [53]	Antibodies to SARS-CoV-2 protect against re-infection during outbreaks in care homes, September and October 2020	2021	209	cohort study	care home residents	prior infection protects against reinfection
Letizia [54]	SARS-CoV-2 Seropositivity among US Marine Recruits Attending Basic Training, United States, Spring–Fall 2020.	2021	3249	cohort study	US Marine recruits	seroprevalence differed by state and ethnic origin
Hall [55]	SARS-CoV-2 infection rates of antibody-positive compared with antibody-negative health care workers in England: a large, multicentre, prospective cohort study (SIREN).	2021	30,625	cohort study	health care workers	previous SARS-CoV-2 infection was associated with 84% lower risk of reinfection: median protective effect 7 months
Gilbert [56]	Immune correlates analysis of the mRNA-1273 COVID-19 vaccine efficacy clinical trial.	2022	1147	phase 3 clinical trial	adults aged 18 and over	neutralizing and binding antibodies were inversely associated with COVID-19 risk
Cohen [57]	Effect of Bamlanivimab vs. Placebo on Incidence of COVID-19 Among Residents and Staff of Skilled Nursing and Assisted Living Facilities: A Randomized Clinical Trial.	2021	1175	randomized controlled double-blind clinical trial	residents and staff of assisted living facilities	Bamlanivimab reduced the incidence of COVID-19 infection
Bar-On [60]	Protection of BNT162b2 Vaccine Booster against COVID-19 in Israel.	2021	1,137,804	cohort study	adults aged 60 years or older	rates of confirmed COVID-19 and severe illness were substantially lower among those who received a booster (third) dose of the BNT162b2 vaccine
Bar-On [61]	Protection against COVID-19 by BNT162b2 Booster across Age Groups.	2021	4,696,865	cohort study	individuals aged 16 years or older	rates of confirmed COVID-19 and severe illness were substantially lower among participants who received a booster dose of BNT162b2
Spitzer [62]	Association of a Third Dose of BNT162b2 Vaccine with Incidence of SARS-CoV-2 Infection Among Health Care Workers in Israel.	2022	1928	prospective cohort study	health care workers	administration of a booster dose was associated with a significantly lower rate of infection over a median of 39 days’ follow up
Menni [63]	COVID-19 vaccine waning and effectiveness and side-effects of boosters: a prospective community study from the ZOE COVID Study.	2022	620,793	prospective cohort study	adults	after 5 months, vaccine effectiveness remained high among individuals younger than 55 years, booster doses restore vaccine effectiveness
**Association with severity and outcome**						
Feng [65]	Correlates of protection against symptomatic and asymptomatic SARS-CoV-2 infection.	2021	171 cases, 1404 controls	data analysis from phase 2/3 vaccine efficacy trial	healthy adults	higher anti-spike IgG, anti-RBD IgG, and neutralizing antibody titers are all associated with lower risk of symptomatic disease
Abu-Raddad [66]	Effect of mRNA Vaccine Boosters against SARS-CoV-2 Omicron Infection in Qatar.	2022	2,293,193	matched retrospective cohort study	population-based study	mRNA boosters led to strong protection against COVID-19-related hospitalization and death
Bar-On [67]	Protection by a Fourth Dose of BNT162b2 against Omicron in Israel.	2022	1,252,331	cohort study	adults aged 60 years or older	rates of confirmed infection and severe illness were lower after a fourth dose of BNT162b2 vaccine
Arbel [68]	BNT162b2 Vaccine Booster and Mortality Due to COVID-19.	2021	843,208	cohort study	adults aged 50 years or older	participants who received a booster at least 5 months after a second dose of BNT162b2 had 90% lower mortality
Mink [69]	Evaluation of SARS-CoV-2 antibody levels on hospital admission as a correlate of protection against mortality.	2023	1152	prospective cohort study	hospitalized adults	anti-SARS-CoV2 spike-antibody levels on hospital admission are inversely associated with in-hospital mortality.

**Table 2:** Indications for anti-SARS-CoV-2 antibody testing. * Comparison of repeat measurements may be necessary to assess the change in antibody levels in individuals who are already seropositive at the time of vaccination.

	Spike (RBD) Antibodies	Nucleocapsid Antibodies
Direct virological testing	-	-
Detect/confirm preceding infection if		
- vaccinated with spike-based vaccine	-	+
- vaccinated with vaccine based on inactivated virus	/	/
- non-vaccinated	+	+
High-risk groups		
- Control of vaccination efficiency in immunosuppressed or oncological patient subsets	+	-
- Monitoring of antibody levels after therapy with monoclonal antibodies	+	-
- Assess protection/need for booster vaccinations in older adults	+	-
- Assess protection/need for booster vaccinations in patients with significant comorbidities and/or severe obesity	+	-
- Proof of effective vaccination for staff working with high-risk groups	+	-
Seroprevalence studies		
- Assess vaccine effectiveness *	+	-
- Estimate proportion of population protected from infection	+	-
- Estimate proportion of population protected from severe courses	+	-
- Estimate proportion of population protected from mortality	+	-

## Data Availability

Not applicable.

## Abbreviations

COVID-19	coronavirus disease 2019
SARS-CoV-2	severe acute respiratory syndrome coronavirus 2
WHO	World Health Organization
BSL	biosafety level
PCR	polymerase chain reaction
RBD	receptor-binding domain
NTD	N-terminal domain
ACE2	human angiotensin-converting enzyme 2
ELISA	enzyme-linked immunosorbent assay
LFA	lateral flow immunoassay
CLIA	chemiluminescent immunoassay
FDA	U.S. Food and Drug Administration
IU	international units
BAU	binding antibody units
CI	confidence interval
aOR	adjusted odds ratio
aHR	adjusted hazard ratio
